# Overcoming challenges of clinical cell therapies for Parkinson’s disease with photobiomodulation

**DOI:** 10.1002/inmd.20240013

**Published:** 2024-07-25

**Authors:** Hossein Chamkouri, Jianmin Si, Peng Chen, Haiyong Ni, Denis E. Bragin, Majid Ahmadlouydarab, Chaoshi Niu, Lei Chen

**Affiliations:** 1 School of Materials Science and Engineering, Hefei University of Technology, Hefei, Anhui, China; 2 Department of Neurosurgery, Division of Life Sciences and Medicine, The First Affiliated Hospital of USTC, University of Science and Technology of China, Hefei, Anhui, China; 3 Anhui Key Laboratory of Brain Function and Diseases, Hefei, Anhui, China; 4 Institute of Resources Utilization and Rare Earth Development, Guangdong Academy of Sciences, Guangzhou, Anhui, China; 5 Lovelace Biomedical Research Institute, Albuquerque, New Mexico, USA; 6 Department of Neurology, University of New Mexico School of Medicine, Albuquerque, New Mexico, USA; 7 Chemical & Petroleum Engineering, University of Tabriz, Tabriz, Iran; 8 Intelligent Manufacturing Institute of HFUT, Hefei, Anhui, China

**Keywords:** dopaminergic neurons, light therapy, neurodegenerative disease, photobiology

## Abstract

Photobiomodulation (PBM) has emerged as a rapidly growing and innovative therapeutic method for various illnesses in recent years. Due to the irreversible nature of Parkinson’s disease (PD), it has proven challenging to impede or postpone the progression of the disease. Despite research on pharmacological approaches to halt neuronal degeneration, the viability of these techniques has been called into doubt due to apprehensions over potential side effects and the ethical implications associated with the utilization of embryonic cell transplantation. Hence, developing an innovative therapeutic approach to halting neuronal degeneration and safeguarding neurons from this neurodegenerative disorder is imperative. This review examines the pathogenesis, challenges, and limitations of conventional PD therapies, allowing a closer examination of PBM’s distinctive approach within this medical context. Delving into PBM’s therapeutic mechanisms in the cells, the effects of different wavelengths on cell therapies in PD patients, and considerations for patient care administration to overcome traditional challenges, this study offers insights into its potential as a promising avenue for PD management.

## 1 | INTRODUCTION

Basal cell therapy is a novel alternative to conventional treatment for neurological disorders, particularly those that cause damage to the nigrostriatal dopaminergic neurons. Due to severe human crises regarding the treatment of Parkinson’s disease (PD), many cell sources were considered for this treatment, but human embryonic cells were ultimately implemented. Even though ethical and logistical issues provided a significant barrier to this work, it is essential to note that in this technique, each individual can only receive an embryo from a helical tissue transplant from their biological group, which confronts numerous issues with cell production. In addition, the treatment is essentially impossible to repeat.^1,2^ The high therapeutic potential of PBM has led to its use in treatment centers for neurological diseases such as PD, which significantly impacts the treatment of these patients.^3,4^ Among these beneficial effects is the ability to heal injured cells including the reproduction of nerve cells and the growth of new cells. PBM is a non-invasive technique for treating brain disorders. Numerous scientists have researched this field and determined that no side effects have been documented during animal research or human treatment.^5,6^ Among the other advantages of light therapy is its ability to eliminate symptoms while simultaneously halting the process of nerve destruction and converting it into the reproduction of nerve cells in the brain.

In contrast, prescription medications can only eliminate symptoms while the destruction of nerve cells persists. Drug therapy is, in most cases, limited to treating symptoms and their lessening; it cannot eradicate the disease, and the expenses of drug therapy can be exorbitant.^7,8^ At the same time, the expenditure of utilizing light-emitting diode (LED) has been substantially lowered, and it offers a high-quality treatment that provides great promise for the future of humankind.^9^

It has been shown that PBM promotes the proliferation of stem cells and their development into dopaminergic neurons. In addition to these benefits, fair treatment improves motor function and reduces inflammation in the neural pathways.^10–12^ Clinical findings suggest that this new approach plays a significant role in establishing an efficient new therapy, although researchers require greater depth and breadth of knowledge of this new method. This review article initially discusses the pathology, challenges, and restrictions of conventional therapy for Parkinson’s patients before turning to the novel approach of PBM in this area of medicine. The new approach considers the effects of the various wavelengths, the therapy procedures, and the administrative aspects of patient care.

## 2 | PATHOLOGY

Examining pathogens across several medical disciplines provides a profound and lucid understanding of illness therapy, such as PD therapy, enabling us to use essential novel therapeutic approaches. At the end of life, PD is characterized by the death of cells in the brain’s basal ganglia, including as many as 70% of neurons that produce dopamine in the substantia nigra. Misfolded alpha-synuclein forms aggregates with other alpha-synucleins, leading to PD. Brain cells cannot clear out these aggregates, so the cytotoxic alpha-synucleins continue to damage brain cells.^13,14^ Lewy bodies are protein clumps that may be observed attached to neurons under a microscope. When neurons in the substantia nigra die, astrocytes (star-shaped glial cells) fall off, and microglia (another form of glial cell) numbers rise steeply. Brock’s staging may be used to gain insight into the neurologic progression of PD.^15,16^ In this model, the olfactory bulb, medulla, substantia nigra, and the rest of the midbrain and forebrain are all thought to be affected by PD. When the substantia nigra is involved, motor signs of PD become apparent.^17–19^

Five main brain pathways connect other brain parts to the basal ganglia and regulate diverse functions. PD affects all five pathways, and their obstruction can explain the disease’s symptoms because these five pathways influence a significant portion of the brain’s functions: counting, learning, attention, and movement.^20,21^ Furthermore, the explorer specifies that an intricate combination of environmental and genetic variables causes PD.^22,23^ About 15% of people with Parkinson’s have a first-degree relative with the illness, and 5%–10% have Parkinson’s resulting from mutations in one of many particular genes.^22,23^ Other risk issues, such as the age of the disease, its severity, and its progression, influence a person’s likelihood of developing Parkinson’s, and the presence of just one of these mutations may not be sufficient to cause the disease.^22,23^

**FIGURE 1:**
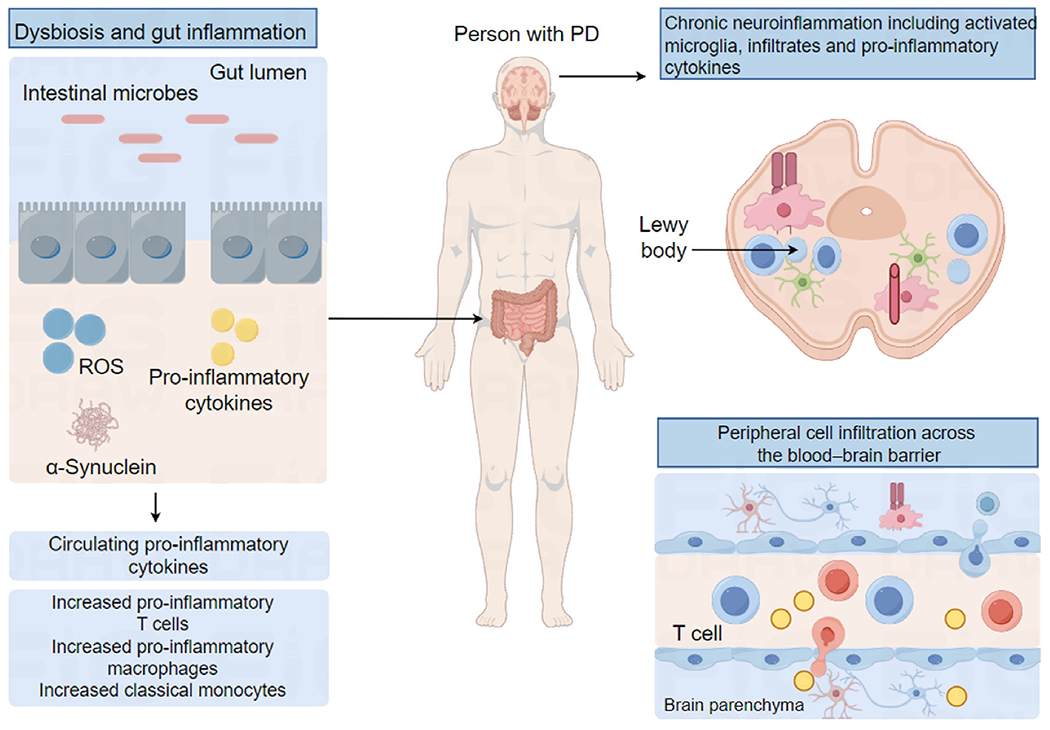
Schematic diagram on the role of innate (the activation of blood-born myeloid cells and microglia) and adaptive (the recruitment of T cells and antibody production) immune behaviors in neuroinflammation.

Several mechanisms, equally inside the degenerating neurons themselves (cell-autonomous) and external the degenerating neurons themselves (non-cell-autonomous), are thought to be involved in the molecular pathways leading to neurodegeneration.^24^ Several factors have been related to the loss of dopaminergic neurons in PD, including mitochondrial dysfunction, alteration of the autophagy-lysosome system, dysregulation of calcium homeostasis, and endoplasmic reticulum stress.^25^ It was hypothesized that environmental variables contributed to the onset of PD when genetic risk factors were identified. Neuroinflammation and immune-mediated pathways have also been hypothesized to play a role. The resident mononuclear phagocytes of the brain, known as microglial cells, are found to undergo consistent classical activation.^26^ Glycosphingolipids (X) are lipids that have a carbohydrate moiety, a ceramide-forming fatty acid chain, and sphingosine. There is considerable variation in the chain length, degree of unsaturation, and hydroxylation status of the fatty acid linked to the sphingosine. Comparable to a lipid (b) but then made of carbohydrates. Sphingolipids, of which ceramides are kind, usually have hydrogen as their X group.^27^ Glucose and galactose may be synthesized from N-acetylneuraminic acid (sialic acid). The names of the many gangliosides, such as asialo, disialo, monosialo, quadrisialo, trisialo, and pentasialo-ganglioside, indicate the wide variety of these compounds.^28,29^ Belarbi et al.^30^ reported about Neurodegeneration at the molecular level in the brain of an individual with PD. The study’s results included modifications to the inflammasome and the secretion of pro-inflammatory cytokines, adjustments in the permeability of the blood-brain barrier, the migration of immune cells from the peripheral regions, and the production of autoantibodies, among several other observations. In addition, several human leukocyte antigen (HLA) variations are associated with the development of PD, including HLA-DRB1 and HLA-DRA. In the lab, PD-causing gene fruits such as alpha-synuclein have been found to activate microglial cells.^29^ In addition to microglia, other cell types, such as macrophages (mature monocytes) and lymphoid cells, could contribute. T cells have been distinguished in the central nervous systems of patients with PD.^30^ This penetration of lymphocytes interested in the brain is not a generic nonspecific leukocyte response, as postmortem human PD brains stained for the cluster of differentiation CD4 (helper T cells) and CD 8 (cytotoxic T cells) but not for CD79 alpha and CD20 (B cells) or CD57 (natural killer cells).^9,10^ It has been revealed that people with PD have wide-ranging deposits of immunoglobulin G on dopaminergic neurons and that these individuals immunolabel Lewy bodies.^10^ However, B cells have yet to be documented as infiltrating lymphocytes despite their conceivable importance being planned. Figure 1 illustrates a schematic of the neuroinflammation and immune-mediated pathways contributing to PD.^31^ It is now known that neurotoxic processes such as decreased neurogenesis, neuronal loss, and altered patterns of synaptic plasticity result from unregulated inflammatory responses and the issue of pro-inflammatory reactive oxygen species and cytokines, even though microglia activation is essential to ensure CNS integrity.^32,33^ Finding targets for modulating immune responses remains a significant obstacle in treating neurodegenerative disorders.^34^ Anti-inflammatory interleukin (IL)-11 cytokines comprise interleukin (IL)-10 and transforming growth factor (TGF)-beta, whereas pro-inflammatory cytokines include interleukin (IL)-1 beta, IL-6, and tumor necrosis factor (TNF)-alpha.^31^ However, many molecular agents may control neuroinflammation. Neuroinflammation has been linked to alterations in glycosphingolipid metabolism in PD and hereditary lysosomal storage diseases.

## 3 | CHALLENGES IN THERAPY

### 3.1 | Challenges of clinical cell therapy

PD is triggered by the death of dopamine-producing cells in the substantia nigra of the brain and diminishes the patient’s motor skills. In recent years, scientists have attempted to utilize the potential of embryonic pluripotent stem cells to generate various tissues. However, cell therapy has always been an obstacle for this and other diseases.^35–37^ Due to ethical debates, researchers are hesitant to use embryonic stem cells, and the use of donated adult stem cells is regrettably associated with the risk of transplant rejection.^38–40^ Autologous stem cells (obtained from the patients) are an alternative option; however, the procedures used to obtain these cells are somewhat invasive and associated with secondary complications.^41–43^ In the case of induced pluripotent stem cells, it should be noted that using viral vectors to transmit Yamanaka factors is problematic. The efficiency of employing these factors in producing stem cells could be much higher.^44,45^ Hospital and clinical data show that people worldwide have undergone these embryo transplants and that each patient’s illness has improved, significantly impacting the disease and their quality of life.^6,7,46^ According to reports from these therapeutic trials, positron emission tomography and postmortem evidence may be kept for over 20 years; however, not all patients can get this transplant.^47^ However, according to some studies, the maximum time frame for taking these therapies is 10 years, and not all patients can benefit from the advantages of this kind of care.^48^ Undoubtedly, the legitimacy of this conduct has raised several inquiries, thereby giving rise to many ethical concerns.

### 3.2 | The challenge of pharmacological therapy

The primary goal of prescription Parkinson’s medications is to treat and manage the condition’s symptoms (Table 1). These medications often work by boosting the dopamine hormone’s activity and release while decreasing acetylcholine levels in the body. The body produces acetylcholine, which interferes with PD therapy.^55,56^ While the absence or demise of brain cells that generate this chemical causes Alzheimer’s or dementia, the pharmaceutical industry has developed several chemical and pharmacological treatments. Still, they have not shown a solid capacity to halt the illness.^57,58^ According to research, the disease’s growth and the process of brain damage persist and have a range of negative impacts on the human body. Levodopa, or L-DOPA, is reportedly recommended to treat early-stage PD by raising dopamine levels.^59,60^ Because some enzymes in the body convert levodopa into dopamine, it effectively treats tremors and muscular stiffness. However, its impact lasts in the body only for 2 hours.^21,61^

Additionally, it is not used for patients with advanced illnesses. Levodopa, in general, aids in the relief of illness symptoms but has little to no impact on the process of limiting the disease’s spread. One further medication that slows the pace at which dopaminergic brain cells are destroyed is Deprenil.^62^ Dopaminergic cells produce and release dopamine, and PD results from degeneration.^62,63^ Deprenil may thus be used to stop the illness from spreading further, but using antidopaminergic medications might negatively affect the patient. Acetylcholine should not be included in medications used by people living with Parkinson’s, according to studies. For this reason, a class of anticholinergic medications that lower the body’s acetylcholine levels is employed.^20,64^ Tremors and muscular stiffness may be effectively treated with these medications. Due to their many adverse effects, these medications should not be taken for an extended period. Chemical medications may temporarily manage and cure illnesses but also have several adverse effects. Levodopa and anticholinergics, two medications used to treat PD, may have adverse effects that affect patients’ ability to swallow food and produce delusions and hallucinations.^60,61,65^ Due to this, other medications are often provided in addition to these to lessen these adverse effects. Additionally, avoiding using anti-Parkinson’s medications just before sleeping is best because medications usually cause side effects such as insomnia, agitation, hallucinations and delusions, osteoporosis, urine incontinence, cognitive problems, and anemia.^66,67^

## 4 | PBM THERAPY

PBM treatment, also known as low-level laser/light therapy, treats biological changes in organisms brought on by photon interactions with molecules in cells or tissues, using the red to near-infrared (NIR) light, which has a 600–1100 nm wavelength.^68^ PBM has shown therapeutic effects against loss of mitochondrial biogenesis or mitochondrial malfunction, the two main routes leading to the development of PD, and a potential target for therapy of microglial activation besides repairing the injured nerve cells, light stimulating neurogenesis and synaptogenesis.^69^

### 4.1 | Neuronal bioenergetics functions of PBM

**FIGURE 2:**
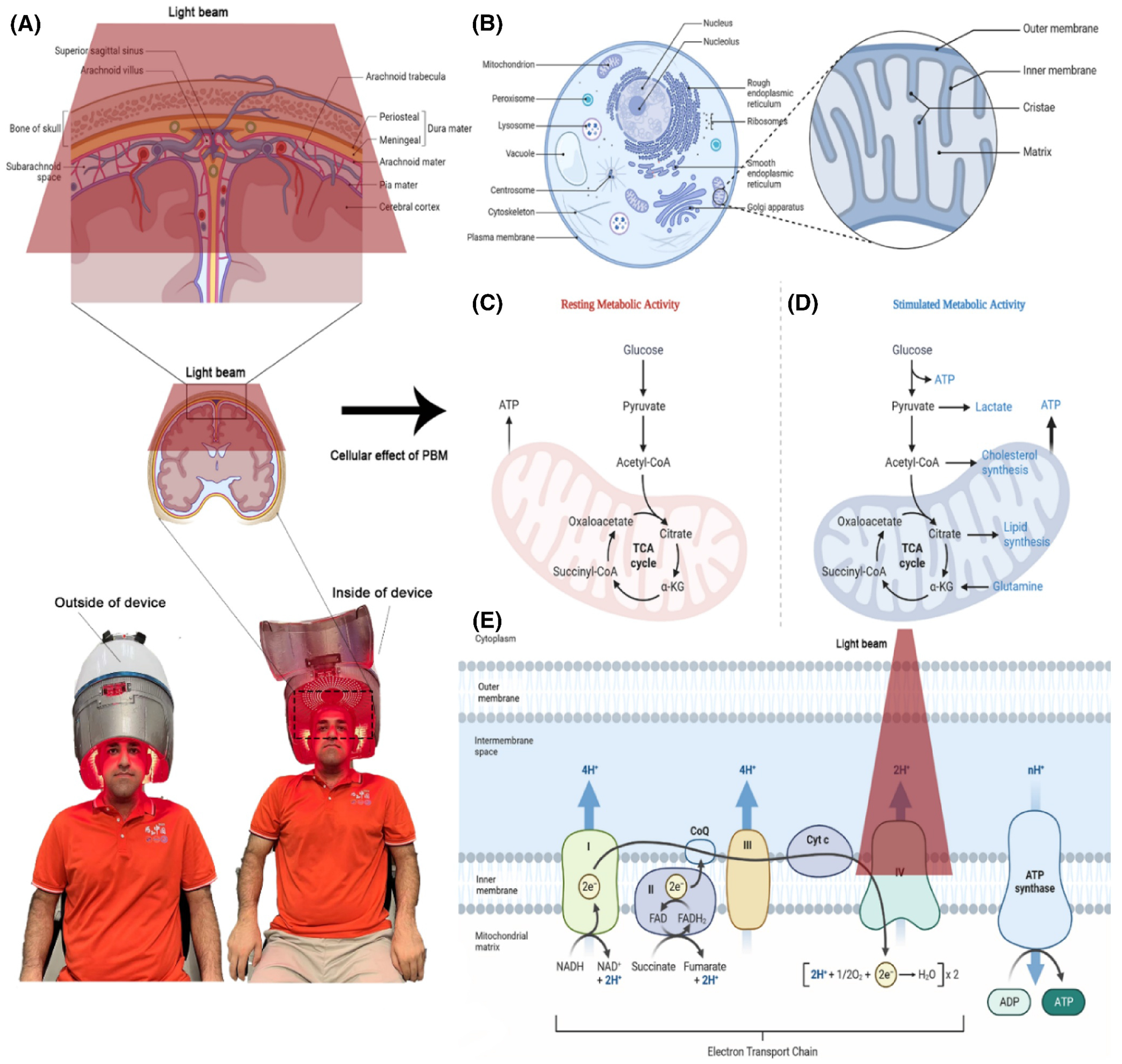
Schematic presence of the molecular mechanism of PBM therapy, illustrated from mitochondria functions. (A) Dr. Hossein Chamkouri, under the supervision of Professor Lei Chen, tested broadband luminescence with wavelengths ranging from 600–1100 nm with adjustable power density; (B) biological structure of the cell with mitochondria as a prominent part of light or PBM therapy; (C and D) resting metabolic activity and stimulated metabolic activity in the mitochondria; (E) the effect of light therapy in the mitochondrial and electron transport chain. The picture was created by biorender.com.

The profound importance of mitochondrial dysfunction in the onset of many neurological and psychiatric disorders has been well acknowledged.^68,69^ Under abnormal conditions, mitochondria can undergo substantial changes, including diminished functioning of the respiratory chain complex and decreased production of ATP, excessive generation of reactive oxygen species (ROS), and the disturbance of mitochondrial membrane potential (MMP), inner mitochondrial permeability transition, and the release of cytochrome c oxidase (CCO) into the cytosol (Figure 2).^69^ Researchers have investigated the beneficial influence of PBM on the energy metabolism of many cell types.^70^ Neural tissue has a significant amount of mitochondria, which renders it vulnerable to interaction with CCO upon exposure to light. CCO serves as an intermediary for brain energy metabolism. This is very important since it is well recognized that the process of PBM in the brain is primarily initiated by the absorption of far-red to near-infrared (NIR) light (600–850 nm) by neuronal CCO.^68–70^

In the initial investigation, Wong-Riley et al. demonstrated a higher rise in CCO activity in cultured rat visual cortex neurons exposed to LED light with wavelengths of 670 and 830 nm and an irradiation dose of 4 J/cm^2^.^70^ This was in contrast to light with wavelengths of 770 and 880 nm. Furthermore, it was shown that the 670 nm light effectively offset the tetrodotoxin-induced decrease in CCO activity. The prefrontal cortex (PFC) of naïve rats showed a 14% increase in CCO activity when exposed to LED light at 633 nm. This rise was seen at 10.9 J/cm^2^ of energy density.

The results were published in the cited work.^70^ Using LED light at the same wavelength increased CCO activity in the superior colliculus by 26% and across the brain by 60% in a rat model of rotenone-induced neurodegeneration. The energy density of the LED light was 3.6 J/cm^2^. It is 108 to the number. Recent work by Zhang and colleagues using transcranial LED stimulation (808 nm) at a dosage of 41 J/cm^2^ has shown a substantial increase in CCO activity in the PFC of a mouse stress model.^71^

Studies examining the maximum cellular ATP synthesis in PBM-exposed cells may provide important insights for improving therapeutic approaches. Research conducted on mouse muscle cells (630 + 850 nm, 2.5 J/cm^2^)^72^ and human brain cells (808 nm, 0.05 J/cm^2^)^73^ has shown that the greatest ATP generation occurred 10 min and 3–6 h after radiation exposure, respectively. Mintzopoulos et al.^74^ recently assessed the cortical levels of Phosphocreatine (PCr) and PCr/β-nucleoside triphosphate (β-NTP) ratios in dogs after acute and long-term transcranial laser treatment (808 nm) using phosphorus magnetic resonance spectroscopy (31P-MRS). The ratios of PCr/β-NTP and PCr levels did not significantly alter after a single radiation exposure. However, 2 weeks of repeated radiation treatment had long-lasting positive effects and enhanced brain bioenergetics. This work^74^ expands on earlier in vitro investigations showing PBM’s transient biostimulatory effects.

In actuality, cells’ own DNA has an impact. Genes undergo mutations under the influence of factors that are responsible for them; sometimes, these are environmental factors and changes in the body, and they issue abnormal orders to produce abnormal proteins, and the DNA of the new proteins has a significant difference in behavior, performance, and tasks^69,70^—leading to neurodegenerative diseases like Parkinson’s. PBM utilizing LEDs selects a specific and compelling wavelength based on the type of cell, cell placement environment, skin color, hair thickness, skull thickness, the profundity of the laser radiation target area, and the patient’s age.^71,72^ Among the observed cellular effects of light therapy are an increase in ATP, the induction of transcription factors, a change in collagen synthesis, an increase in blood flow, and the stimulation of angiogenesis.^73,74^ In the past few years, 18 fact sheets have reported on using LED light in the skull and brains of animals, while only 11 articles have used lasers as a light source to treat PD.^75^

Moreover, 26 studies used red and far-red wavelengths to treat Parkinson’s.^73–75^ In the cited studies, the wavelengths 670 nm, 808 nm, and 675 nm were employed in approximately 21 studies, four studies, and two research works, respectively, whereas 405, 627, and 630 nm wavelengths were used only once each.^73–75^ Red light has been utilized in most of these studies not because it is superior to light with a wavelength of 810 nm but because of its ability to affect the eye and heal wounds. According to these reports, the treatment intensity in these extensive studies has typically ranged between 20 and 50 mW/cm^2^. In fact, by stimulating and influencing the cell’s metabolism, these wavelengths normalize the cell’s behavior. Moreover, it is noticeable that Age-related neurodegenerative disease Alzheimer’s disease (AD) affects worldwide socio-economic and healthcare systems. Limited therapeutic options for AD need creative techniques to improve amyloid β (Aβ) protein removal and cognitive capacities. PBM is a non-invasive and effective therapy for numerous brain illnesses. Li et al.’s study shows that 1267-nm PBM reduces cognitive impairment in the 5 × FAD mouse model of AD without raising brain temperature. They found that PBM reduces Aβ plaques differently in different prefrontal cortex and hippocampus subregions by employing 3D tissue optical clearing imaging and automated brain region segmentation.^75^ In mice with 5 × FAD, PBM-induced lymphatic clearance of Aβ has been associated with improved memory and cognitive performance.^75^ Their data suggest that regulating meningeal lymphatic vessels (MLVs) may significantly affect Aβ clearance.^75^ This pilot research shows that PBM can improve Aβ elimination in mice with 5 × FAD brains.^75^ This improves the neurocognitive status of rats with AD, suggesting that PBM may be a feasible and practical AD therapy.^75^

## 5 | CLINICAL STUDY

From a medical perspective, different brain areas are affected by a broad spectrum of neurological and psychological illnesses. Recent animal-models study and clinical brain PBM treatment trials have tried to address PD.^10,76–98^ Moreover, there is also rising interest in using this noninvasive technique to treat these patients due to its cost-effectiveness, high ability to regenerate brain nerve tissues, long-lasting treatment alternatives, low possibility of side effects, and the possibility of being administered at home by elderly patients.

### 5.1 | Animal study

Researchers and studies have been experimenting with PBM in mouse models of PD for several years and decided to see if PBM directed at the body might shield the brain (Table 2). Notwithstanding our satisfaction with the outcomes, scientists know that PBM has drawbacks.^83,84^ The concept that PBM delivered to a peripheral tissue that is simpler to access than the brain may activate systemic defenses that shield distant areas piqued our curiosity.^83,84^ Researchers knew, however, that transmitting a signal from the periphery to the brain is challenging due to the blood-brain barrier (BBB).^85,86^ One assumption they made—which may not have been accurate—was that distant PBM-encouraged neuroprotection is likely brought on by circulating substances that would be essential to penetrate the BBB to do so.^85,86^ To determine if distant PBM had neuroprotective benefits, scientists conducted a “helmet” experiment. The animal’s back was illuminated in this experiment, while aluminum foil was placed over its head. They employed a mouse model of early PD brought on by an intraperitoneal injection of the neurotoxic 1-methyl-4-phenyl-1,2,3,6-tetrahydropyridine (MPTP). As a per-conditioning intervention, researchers exposed the animal to a 670 nm LED light (50 mW/cm^2^) on its back or head.^12,86^ On each MPTP injection day, PBM was administered twice daily (4 J/cm^2^ of radiated light per action). Following MPTP injections, mice were permitted to survive for a whole week. The World Association for Laser Therapy Convention shared some early findings from the Helmet research.^87–89^ This first experiment involved injecting 50 mg/kg MPTP into mice over 2 days, with the primary result ratio being the quantity of efficient dopaminergic cells in the substantia nigra pars compacta (SNc) as characterized by tyrosine hydroxylase (TH) stereological and immunolabelling analysis.^12,90^ As anticipated, MPTP caused the SNc to lose 35%–40% of its dopaminergic cells. According to their preliminary data, transcranial PBM significantly protected these cells from damage. Mice who received PBM to the head had cell counts comparable to those of controls who received saline injections. The most startling outcome, however, was seen in mice that received PBM that was strictly directed at the body; even though the head was not directly exposed to radiation, these mice displayed meaningfully developed SNc dopaminergic cell amounts than sham-cured MPTP animals, demonstrating that PBM of the body protects the brain.

**FIGURE 3:**
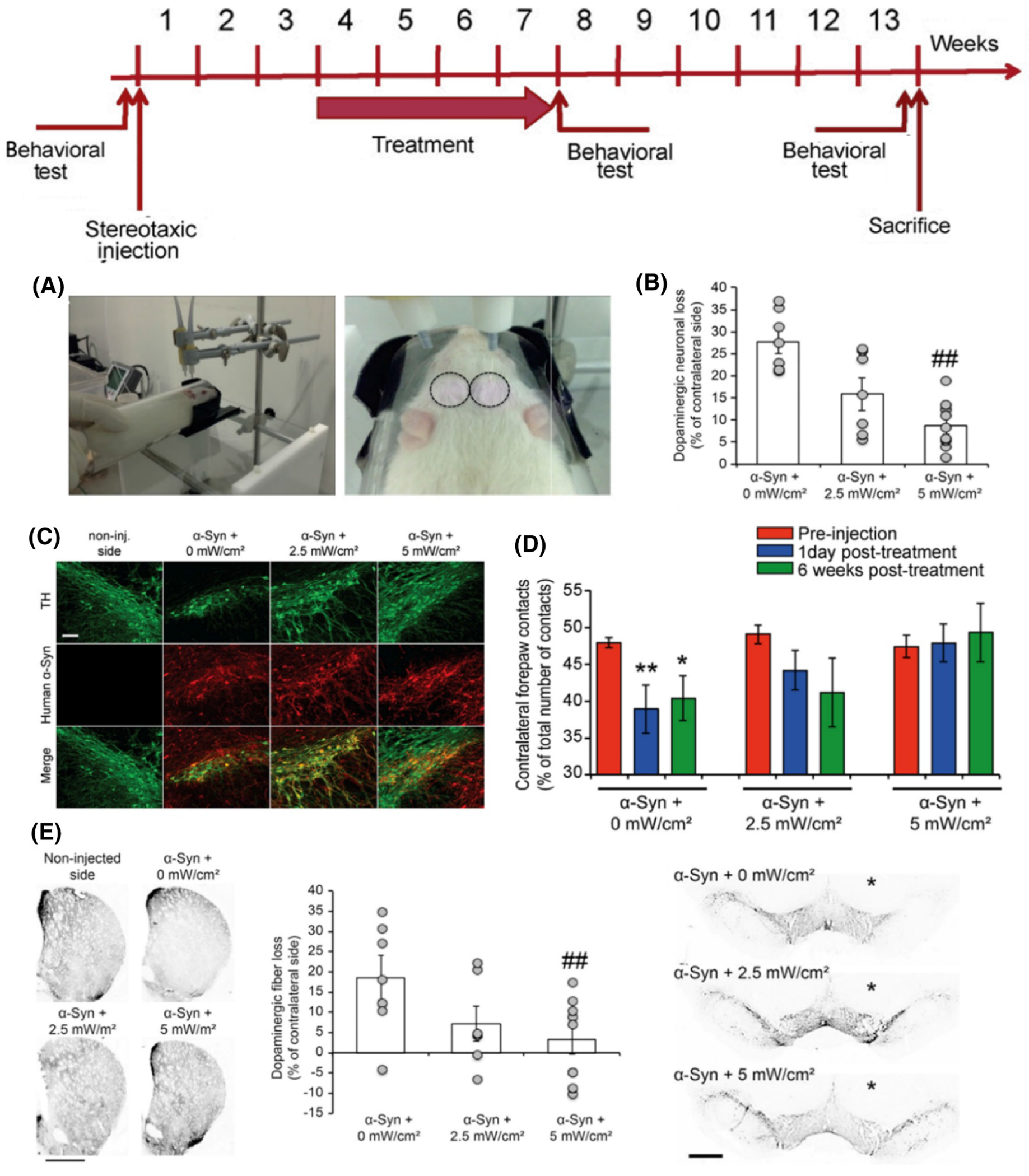
Results of animal research experiment at the Johnson & Johnson company,^88^ denoted with the experimental chronology, technique, and NIR light application. (A) The rats in a clear plexiglas cylinder and circles delineating the head surface from the eyes and ears, (B) long-term use of PBM reducing the loss of dopaminergic neurons produced by α-syn, (C) no significant variation been observed in the expression levels or pattern of human α-synuclein in nigral TH + dopaminergic neurons between experimental conditions after 3 and 6 weeks of abstinence, (D) long-term use of PBM reducing motor impairments caused by α-synuclein (D), long-term exposure to PBM minimizing the loss of dopaminergic nerve fibers in the striatum caused by α-synuclein (E). Reproduced under terms of the CC-BY license.^88^ Copyright 2015, The Authors, published by PLOS.

A more extensive, inclusive helmet research was released in 2014.^91^ More animals, glial cells, and MPTP dosage totals were included. As determined by TH immunohistochemistry, this study has shown that transcranial PBM and distant PBM offer significant neuroprotection against PD insult when combined with 50 mg/kg MPTP.^91^ The number of cells in the two groups did not significantly differ, but in this model, transcranial PBM seemed to preserve neurons more effectively than distant PBM. Neither transcranial PBM nor far-off PBM could halt the harm MPTP produced to the SNc at higher dosages (75 and 100 mg/kg). Also, scientists counted the quantity of microglia and astrocytes in the SNc of mice administered 75 mg/kg MPTP by glial fibrillary acidic protein (GFAP) and immunohistochemical labeling of ionized calcium-binding adapter molecule 1 (IBA1). MPTP poisoning significantly increased the number of IBA11 cells, which neither transcranial nor distant PBM could decrease, even though it did not affect GFAP labeling substantially.^92^ One issue with the abovementioned trials was the concurrent use of PBM (remote or transcranial). This raises the possibility that the observed benefits were brought on by PBM interfering with the body’s ability to produce MPTP rather than by neuroprotection brought on by PBM. To address this issue, the researcher conducted research in which far-off PBM was applied as interference pre-conditioning. For 2, 5, or 10 days, mice were exposed to 670 nm LED light (50 mW/cm^2^) once daily (4 J/cm^2^ of light emission for each action). At least 24 h they had passed after the final remote PBM treatment before any groups began receiving MPTP injections (an overall of 50 mg/kg). Pre-conditioning through far-off PBM for 10 days seems to reduce MPTP-encouraged damage of dopaminergic cells in the SNc and connected aberrant neuronal action in the caudate-putamen intricate, according to examination using FOS immunohistochemistry.^17^ These first observations support the hypothesis that PBM in exterior tissues can shield the brain. Moreover, Researchers discovered that extended PBM treatment diminishes the degeneration of dopaminergic neurons induced by the excessive production of human α-synuclein in the substantia nigra of a rat model with PD.^88^ The method included exposing both sides of the rat’s head to 808-nm near-infrared light daily for 28 days, which enhanced motor function affected by α-syn, as assessed using the cylinder test. This medicine significantly reduced the loss of dopaminergic neurons in the injected substantia nigra and preserved dopaminergic fibers in the ipsilateral striatum. The beneficial effects remained for at least 6 weeks after the treatment ceased. Their data indicate that PBM might be a promising treatment strategy for addressing PD and other synucleinopathies.^88^ A study experiment conducted on animals at the Johnson & Johnson business yielded the following results.^88^ In this illustration, the experimental chronology, procedure, and application of NIR light are shown. The rats are contained inside a transparent Plexiglas cylinder. Circles are used to differentiate the head surface from the eyes and ears (Figure 3A). Long-term usage of PBM has been shown to prevent the loss of dopaminergic neurons that are induced by α-synuclein (Figure 3B). There was no discernible difference seen in the amounts of human α-synuclein expression or the pattern of its expression in nigral TH + dopaminergic neurons between the experimental conditions after 3 weeks and 6 weeks of abstinence (Figure 3C). Using PBM for an extended period may help lessen motor deficits that α-synuclein causes (Figure 3D). Additionally, prolonged exposure to PBM can help limit the loss of dopaminergic nerve fibers in the striatum that is caused by α-synuclein (Figure 3E).

### 5.2 | Human study

Human models and case studies may be used to demonstrate the efficacy of PBM as an additional therapy for PD symptoms. Liebert et al.^93^ gathered 12 patients with idiopathic PD. A 12-week course of PBM, including transcranial, intranasal, neck, and belly approaches, was administered to six randomly selected volunteers. The other six patients were placed on a 14-week waiting list before receiving the same therapy. They offered each participant a PBM gadget to assist them in continuing their treatment outside of the 12-week session. The participants’ mobility, fine motor skills, balance, and cognitive abilities were assessed at baseline, four weeks, 12 weeks, and after the home therapy session. Furthermore, evaluations were performed before the commencement of treatment. The efficacy of the therapy was evaluated using a Wilcoxon Signed Ranks test at a significance level of 5%.^94^ Additionally, they noticed substantial improvements in mobility, cognitive function, dynamic balance, and fine motor function (*p* < 0.05) from 12 weeks to a year of PBM therapy. The participants believed that the statistically significant clinical improvement was impressive despite the many individual advantages. Even though each person’s path to recovery was different, many continued to progress even after a year of home treatment. There was a noticeable Hawthorne Effect in addition to the treatment’s effects. It was discovered that the treatment had no unfavorable side effects. After analyzing the literature, they concluded that PBM offers promise as a safe and effective therapy for several PD symptoms and clinical presentations. With a neurological illness where deterioration is often expected, and treatment is consistently administered, up to a year of continuous improvement has been seen. Patients with PD may benefit from home care alone or with a caretaker. The findings of this study need further research using a larger RCT.^94^

**FIGURE 4:**
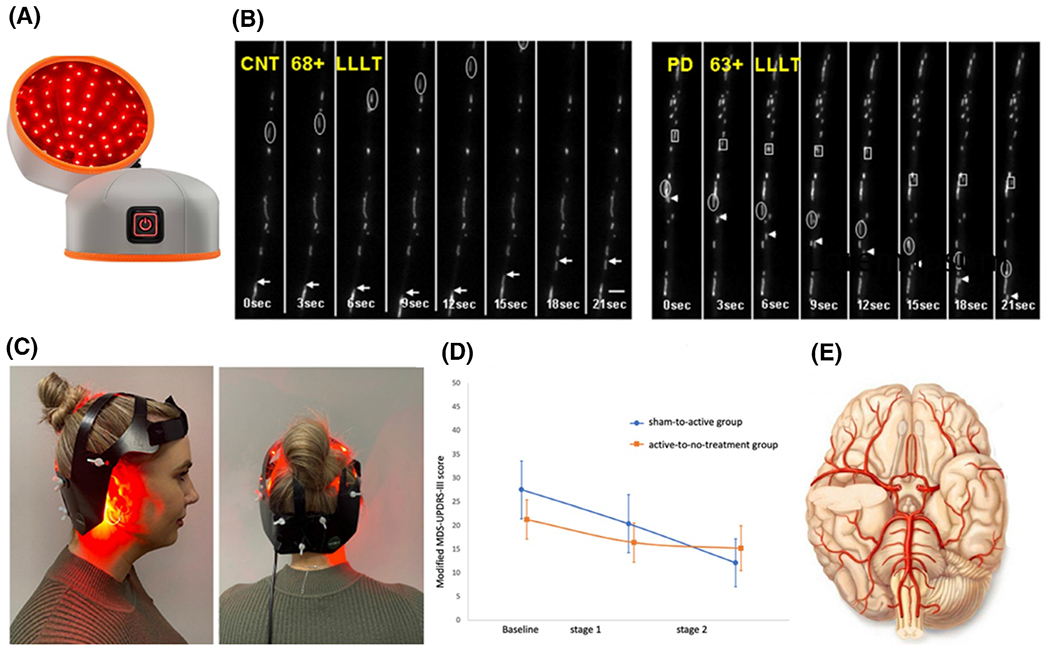
The CerebroLite and “Neuro” helmet PBM devices. (A) Image of PBM helmet for therapy, (B) the Montages showing the mobility of mitochondria tagged with MTRed during CNT68, CNT68-90 min post-LLLT, PD63 min, and PD63-88 min post-LLLT.^95^ (C) the PBM “Neuro” helmet gadget, viewed from the side and back,^101^ (D) the comparison of treatment outcomes in sham-to-active and active-to-no-treatment groups over 24 weeks,^101^ (E) schematic representation of enhanced cerebral blood flow after light therapy. Reproduced under terms of the CC-BY license.^95^ Copyright 2009, The Authors, published by Springer Nature. Reproduced under terms of the CC-BY license.^101^ Copyright 2023, The Authors, published by Elsevier.

Although the clinical data on the treatment of PD with PBM are not ample, all the data show positive therapeutic effects and no side effect was observed (Table 3). The patient is doing well according to the early findings of this innovative therapy approach. More comprehensive information on treatment protocols and symptom modifications may be found in the lengthy clinical studies,^99,100^ which contrast the pre- and post-light therapy states of Parkinson’s patients. This case study examines how a PBM helmet affects individuals exhibiting motor impairment symptoms (Figure 4A). The study focused on how medication affected each patient’s unique symptoms and how motor skills (including writing and analysis) changed. After receiving cranial light treatment, every single one of the patients has experienced notable, although slight, improvements in their overall health and everyday functioning. According to the findings, 75% of people had improvements in their initial PD symptoms and indicators, whereas 25% saw no change at all.^99,100^ Furthermore, there was no evidence of worsening symptoms in the clinical research. Undoubtedly, PBM—rather than Dicher’s—was the crucial therapeutic element. Three patients continued to use the same dose of medicine throughout therapy, whereas one patient dramatically decreased their dosage. These patients have reported improvements in their non-physical and physical complaints following PBM treatment. Two individuals had their abnormal gait and tremors addressed. Furthermore, a second patient’s sleeping patterns improved.^99^ Because of PBM’s various advantages, which have helped relieve various ailments (although varying degrees depending on the individual), patients have experienced a better quality of life. The trial participants did not experience any adverse effects or deterioration of their health while undergoing therapy.^99,100^ The second indirect tactic PBM uses is light-mediated regulation, which regulates the immune system.^101^ PBM suppresses inflammation caused by cytokines, lessens inflammation on nerves, and controls the function of damaged cells by inducing the immune system.^102^ The primary mechanism described in the study’s findings is indirect stimulation under severe conditions. Previous studies have shown that the brain stem and basal ganglia, the primary areas affected by neuronal growth in PD, are not responsive to light treatment.^78,103^ Studies on animals have shown that light therapy may boost immunity by modifying the skin’s outer layer, accessing deeper tissues, and affecting human blood vessels and circulation patterns. Radiation exposure to the brain’s frontal areas changes blood flow and significantly impacts immune cells (Figure 4B).^95,104–106^ A patient who received therapy saw a resurgence of Parkinson’s symptoms due to a potent strain of influenza virus. Amazingly, all indications of PD vanished when the condition became better. The findings of this study suggest that the patient’s immune system was compromised by influenza, which is how they became ill.^106^ As a consequence, the phototherapy target cells in the brain were able to respond anti-inflammatorily to specific wavelengths. The sickness is over and has accomplished its goal. The immune system and the neurological system are essential to this therapeutic strategy. Herkes et al.^101^ set out to investigate the safety and effectiveness of transcranial photobiomodulation (tPBM) to reduce the motor symptoms associated with PD. A randomized, controlled, double-masked technique was used in this study to assess its feasibility. The participants in this research were 59 to 85-year-olds who had idiopathic PD. They received 72 sessions of treatment utilizing a PBM helmet over 12 weeks. During these sessions, the participants received active or sham therapy as part of the trial’s first phase. Zoom online video conferencing was used to supervise the intervention while the participants were home. Following stage 1, participants were divided into two groups: the active-to-no-treatment group, which received no treatment for 12 weeks, and the sham-to-active group, which received active therapy for 12 weeks. The dynamic helmet system emits red and infrared light over the head for 24 minutes, 6 days a week. Ten men and 10 women comprised 20 participants who were allegedly randomly allocated to two groups between December 6, 2021, and August 12, 2022. The active group as a whole completed a 12-week program, while the sham group had treatment for 18 weeks. In the control group, 12 participants finished the full 12 weeks of active therapy, whereas 14 others opted out of treatment and stayed in the sham group. The medication was highly accepted, simple to use, and had few unfavorable short-term side effects. Of the nine likely adverse events reported during the active treatment phase of the trial, two mild responses could have been connected to the device in the group that switched from the sham treatment to the active therapy. One person’s leg muscles were momentarily weaker. The fine motor skills of the participant’s right hand decreased in dexterity. They both proceeded to the experiment. Their research contributes to the increasing data suggesting that targeted tPBM might be a non-pharmaceutical, safe, and effective adjuvant treatment for PD (Figure 4C). Further study is required, but the findings provide the foundation for a reasonable randomized placebo-controlled clinical trial. The clinical experiment has several restrictions. In light of the number of participants who left the sham-to-active group, the research initially lacked sufficient power to be regarded as a feasibility experiment. Consequently, there is little opportunity for study and understanding, and prejudice may impact the outcomes. The necessity for caregivers, the lack of space for online tests, and the computer proficiency requirements might have introduced bias into the participant selection processes. Age-related neurodegenerative disease AD affects worldwide socio-economic and healthcare systems. Limited therapeutic options for AD need creative techniques to improve Aβ protein removal and cognitive capacities.

PBM is a non-invasive and effective therapy for numerous brain illnesses. A study^75^ shows that 1267-nm PBM reduces cognitive impairment in the 5xFAD mouse model of AD without raising brain temperature. Their work found that PBM reduces Aβ plaques differently in different prefrontal cortex and hippocampus subregions by employing 3D tissue optical clearing imaging and automated brain region segmentation. In mice with 5xFAD, PBM-induced lymphatic clearance of Aβ has been associated with improved memory and cognitive performance. Their data suggest that regulating meningeal lymphatic vessels (MLVs) may significantly affect Aβ clearance. This pilot research shows that PBM can improve Aβ elimination in mice with 5xFAD brains. This improves the neuro-cognitive status of rats with AD, suggesting that PBM may be a feasible and practical AD therapy.

## 6 | HEALTHCARE AND ECONOMIC TRENDS

Cell treatments represent a significant step forward in the treatment of PD. However, it should be emphasized that combining cell therapies utilizing photons and light therapy is a new technology showing great promise. Despite this, many challenges still need to be mastered in the centers, and this can only be done with correct and precise strategies. It offered solutions to issues that arose in research, medical care, hospitals, and clinical trials.^19,89,107^ Cell therapy was ineffective for many people before the advent of PBM. The advent of PBM as the latest human technology gave patients new optimism; however, many questions remain unanswered, including the definitive relationship with cells. In a broad sense, fair treatment makes intracellular metabolism more robust and consistent.^73,108,109^ Furthermore, does universal neuroprotection exhibit a specific preference for peripheral tissue as its target, or is this preference contingent upon the particular type of brain disorder? Which wavelength, irradiance, and treatment frequency are optimal for inducing neuroprotection in far-off PBM procedures? Is it possible to customize treatment programs based on the individual body composition of each patient? Does the process of aging impact our capacity to react to distant PBMs? What mechanisms are underlying neuroprotective effects caused by foreign PBMs? Moreover, might understanding these mechanisms help create biomarkers for therapy effectiveness?

In contrast to laborious and intrusive surgical procedures, it is an entirely non-invasive approach that does not cause any adverse effects. Most importantly, the cost of this Parkinson’s therapy approach is far cheaper than other Parkinson’s treatment methods.^110–112^ It is an excellent technique since it utilizes light treatment and has a high potential of photons to target a particular location with an injury without posing any risks. Although several investigations have shown that the chance of experiencing adverse effects from this medication is relatively low, it is only natural that they will one day better comprehend the risks associated with using it.^10,113,114^ When dealing with cells and the changes that occur inside of cells and genetic alterations, it is recommended that future studies pay more attention to several wavelengths across various periods. Patients suffering from neurological and brain conditions have reason to be optimistic about the future owing to this technology, which is economical and straightforward to use in both clinical and domestic settings.^113,114^ The method of manufacturing the gadget is yet another significant aspect of the present investigation. It is essential for the optical equipment to be light and simple to adjust so that elderly individuals may use them without difficulty.^115,116^

## 7 | CHALLENGES IN DEVELOPING PBM DEVICES

The primary obstacle is our inability to determine the region of the human body that is most efficient. In previous studies, as mentioned in this review article, researchers believed that light should be directed toward the neurons in the brain, and their primary focus was on the advancement of transcranial PBM.^93–100^ A recent study demonstrated the efficacy of remote PBM targeting the belly or legs.^117^ Hence, it is crucial to prioritize fundamental investigations into light photophysiology to create effective PBM devices that elucidate the mechanics of PBM photobiology. The advent of the fourth industrial revolution has heightened recognition regarding the imperative to incorporate the vast network of connections between humans and technology. Academic researchers are developing a formless apparatus that offers various advantages, including wearability, flexibility, disposability, and adaptability. Before incorporating light-emitting materials into body-conformable items, it is imperative to address several pertinent concerns. There is a need for enhancement in the mechanical properties of light-emitting materials. Inorganic light emitters exhibit a lack of flexibility and rigidity. Structural design techniques using rigid islands, buckling, and inherently stretchy nanoparticle-polymer-matrix can enhance stretchability. Nevertheless, these proposed techniques can potentially diminish optical performance and resolution. Organic light-emitting materials have greater flexibility, yet their viability is still pending significant advancements. Nanoconfinement is a promising method for enhancing the luminosity and flexibility of light-emitting polymers, although its suitability may vary. Regrettably, existing light-emitting polymers cannot undergo self-repair or degradation, rendering them inappropriate for use in skin or implants. The limited mobility of light-emitting polymers impedes charge transfer. Impracticality arises when highly ordered light-emitting polymers are used to generate high-resolution displays on film or soft fiber surfaces. In contrast to inorganic materials, organic light-emitting materials exhibit lower stability, imposing constraints on their operational longevity.

## 8 | CONCLUSIONS

Within the extensive field of PBM research, the most effective approach has been “direct PBM,” which involves directing light toward the specific tissue one seeks to modify or preserve. Nevertheless, the emergence of “remote PBM” presents a viable solution to address the limitations associated with light penetration into deep body tissues, specifically the brainstem, basal ganglia, thalamus, and midbrain. This approach utilizes the indirect effects of PBM by directing light toward one biomatter to protect others. Several unresolved issues persist as the investigation of neuroprotection using distant remote PBM is still in its early stages. Despite some unresolved difficulties, remote PBM represents a promising advancement due to its ability to satisfy multiple requirements. These criteria include security, simplicity, painlessness, non-invasiveness, affordability, user-friendliness, and patient tolerance. The perspective of far-off PBM highlights the intricate nature of animal physiology. This underscores the importance of understanding previously unidentified relationships between physiological tissues in pursuing effective neuroprotective treatments and their possible therapeutic applications. The initial hurdle is our inability to determine the precise region of the human body that demonstrates the greatest effectiveness. Historically, researchers had the opinion, as mentioned in this review article, that it was imperative to direct light onto the neurons in the brain with the primary goal of advancing transcranial PBM. Recent research has demonstrated the efficacy of remote PBM in specifically targeting the stomach region or legs.^117^ Hence, it is crucial to prioritize fundamental research on the photophysiology of light to facilitate the development of effective PBM devices capable of elucidating the mechanisms underlying PBM photobiology. The procedure is highly effective as it employs light therapy and many photons to precisely target a specific injured area without presenting any danger. While multiple studies mentioned above have indicated a relatively low likelihood of encountering negative effects from this medicine, they will inevitably have a more thorough understanding of the hazards involved with its usage. Before integrating light-emitting substances into body-conforming objects, it is crucial to address many relevant challenges. Improvements are required in the mechanical characteristics of light-emitting materials. Inorganic light emitters lack both flexibility and stiffness. Using rigid islands, buckling, and nanoparticle-polymer-matrix with intrinsic stretchiness can improve the ability to stretch in structural design procedures. The future of PBM in treating different neurological illnesses, such as PD, is promising and will bring new hope to human civilization.

## Figures and Tables

**TABLE 1:** Common medications for PD with their side effects.

Name of drugs	Functionality	Side effect
A combination of levodopa and carbidopa^49, 50^	It is one of the drugs used in PD. Carbi Dopa helps levodopa work better. Therefore, the dose of levodopa can be reduced.	They reduced nausea, vomiting, and erratic heartbeat. Sinmet’s short-term adverse effects are the lowest of this disease’s medications. Longterm usage may cause involuntary movement. Take levodopa inhalation powder and estradiol pills between doses to manage symptoms. After using levodopa for 3–5 years, users may develop adverse effects such as restlessness, disorientation, or involuntary movements. These adverse effects may be avoided by changing dosage duration. Safinamide medicines are provided to those with symptoms of illness following symptom management with levodopa and carbidopa. It seems that adding this medicine to the others will prolong symptom relief. Sleep difficulties, nausea, falls, and uncontrolled movements are common adverse effects.
Dopamine agonists^51^	These medications mimic brain dopamine. It will include ropinirole and pramipexulfo rotigotine. You may take Cinmet with or without these drugs. Some clinicians administer dopamine agonists first, then levodopa for unmanageable symptoms.	Long-term levodopa is riskier than dopamine agonists. Thus, PD therapy begins with it. Short-term use of these medications might cause nausea, vomiting, dizziness, headache, light sensitivity, disorientation, and hallucinations.
Anticholinergic drugs such as Benztropine and trihexyphenidyl^52^	These medications normalize dopamine and acetylcholine levels and alleviate tremors and muscular stiffness.	Drugs like these damage memory and thinking, particularly in elderly folks. Therefore, physicians utilize this drug less nowadays.
Mao-B inhibitors such as selegiline and rasagiline^53^	These medications suppress toxic dopamine molecules in the brain, improving brain function. Some data suggests selegiline reduces brain disease development, particularly early on.	Side effects of this medication include nausea, dizziness, lethargy, and stomach discomfort. Animal studies indicate rasagiline slows disease development. Headache, joint discomfort, dyspepsia, and depression are adverse effects. COMT inhibitors: Entacapone, capone, tolcapone. COMT deactivates levodopa after use. COMT inhibitor medicines improve levodopa usage and lessen symptoms by inhibiting COMT.^54^

**TABLE 2:** A brief investigation of PBM therapy influence on PD animal models.

Title	Irradiance	Therapy	Irradiation time per session	Improvements
Near-infrared light therapy protects midbrain dopaminergic cells in MPTP-treated rats^76^	40 mW/cm^2^ (at scalp)	Near-infrared light treatment, 670 nm	90 s	Neuroprotection of dopaminergic cells in the substantia nigra pars compacta (SNc) and the zona incerta-hypothalamus (ZI-Hyp) from degeneration in MPTP-treated mice.
Survival of dopaminergic amacrine cells after near-infrared light treatment in MPTP-treated mice^77^	40 mW/cm^2^ (at scalp)	Near-infrared light treatment, 670 nm	90 s	Survival of dopaminergic amacrine cells in the retina in MPTP-treated mice
Patterns of cell activity in the subthalamic region associated with the neuroprotective action of near-infrared light treatment in MPTP-treated mice^78^	40 mW/cm^2^ (at scalp)	Near-infrared light treatment, 670 nm	90 s	Changes in patterns of Fos expression in the subthalamic region (subthalamic nucleus and zona incerta) after near-infrared light treatment in MPTP-treated mice
PBM enhances nigral dopaminergic cell survival in a chronic MPTP mouse model of PD^79^	40 mW/cm^2^ (at scalp)	Near-infrared light treatment, 670 nm	90 s	Enhanced survival of dopaminergic cells in the substantia nigra pars compacta (SNc), periaqueductal gray matter (PaG), and zona incerta-hypothalamus (ZI-Hyp) in a chronic MPTP mouse model of PD
A transgenic Parkinsonian mouse model’s dopaminergic cell survival after near-infrared light exposure^80^	40 mW/cm^2^ (at scalp)	Laser, 808 nm	100 s	Oxidative stress reduction and hyperphosphorylated tau expression in a transgenic mouse model of Parkinsonism.
Transcranial PD (mouse MPTP model), 1 cm from head^81^	40 mW/cm^2^ at scalp, 5.3 mW/cm2 inside skull, 0.47 J/cm^2^ per season	LEDs, 670 nm	Four seasons over 30 h, 90 s, irradiation area of 10 cm^2^	SNc TH + cell growth with 50 and 100 mg/kg MPTP
Hold probe 1–2 cm from the head for transcranial PD (MPTP Balb/c and C57BL/6 mice models)^78^	0.47 J/cm^2^ per irradiation	LEDs, 670 nm	4 irradiations over 30 h, 90 s	Balb/c mouse: Increased SNc TH + cell counts and OFT locomotor activity, including high mobility, velocity, and immobility periods.
PD (mouse MPTP models), head or body irradiation^82^	40 mW/cm^2^, 4 J/cm^2^	LEDs, 670 nm	90 s	At body or head radiations, SNc TH + cell accounts and glial cell statistics improved at 50 mg/kg MPTP and 75 mg/kg MPTP, respectively.

**TABLE 3:** A brief investigation of PBM therapy influence in human PD models.

References	Effect	Models	Parameters	Source
Trimmer et al.^95^	The average velocity of mitochondrial movement in PD cybrid neurites was significantly increased and restored to levels comparable to CNT.	PD and disease-free age-matched volunteer controls (CNT)	810 nm, 50 mW/cm^2^ for 40 s	Laser
Quirk et al.^96^	Enhanced mitochondrial ability besides oxidative stress	In vitro (human)	670 nm, 40 mW/cm^2^, 14.4 J/cm^2^ over 30 h	LED
Maloney et al.^97^	Enhanced stability, freezing, gait, rolling in bed, cognitive function, and problems in dialogue were evaluated through the visual analog scale	In vivo (human)	Daily for 2 weeks	Laser
Hamilton et al.^84^	55% of the initial signs and symptoms of the six patients showed overall improvement, whereas 43% stayed the same and only 2% got worse.	Six patients with Parkinson’s disease that used in-house built photobiomodulation (PBM) helmets.	Used “buckets” lined with light-emitting diodes (LEDs) of wavelengths across the red to near-infrared range (i.e., 670, 810, and 850 nm; *n* = 5) or a homemade intranasal LED device (660 nm; *n* = 1)	LED
Liebert et al.^93^	The mobility, cognition, dynamic balance and fine motor skill were significantly improved (*p* < 0.05) with PBM treatment for 12 weeks and up to 1 year. More importantly, no side effects of the treatment were observed.	Twelve participants with idiopathic PD were recruited. Six were randomly chosen to begin 12 weeks of transcranial, intranasal, neck and abdominal PBM. The remaining 6 were waitlisted for 14 weeks before commencing the same treatment.	Administered transcranially with a VieLight Neuro Gamma device (4 LEDs, 240 J), intranasally with a VieLight Gamma nasal device (1 LED, 15 J), transdermally to the C1/C2 region of the neck and to the abdomen with an Irradia MID 2.5 laser device (4 laser diodes, 39.6 J) or a MIDCARE laser device (2 diodes 39.6 J).	PBM device
Liebert et al.^98^	A number of clinical signs of PD, including mobility, cognition, dynamic balance, spiral test, fine motor skill, and sense of smell, were shown to be improved by remote PBM treatment.	Seven participants were treated with PBM to the abdomen and neck three times per week for 12 weeks.	A laser device with four class-1 diodes (904 nm 30 mW) targeting nine abdominal points (1 min each), and the posterior C1/C2 region of the neck (1 min), to give a total abdominal dose of 64.8 J and a neck dose of 7.2 J (Figure 1)	Laser
Herkes et al.^101^	The transcranial PBM (tPBM) treatment is safe and is feasible to be delivered as a non-pharmaceutical adjunct therapy for Parkinson’s disease.	Double-blind, randomised, sham-controlled feasibility trial, patients (aged 59–85 years) with idiopathic Parkinson’s disease were treated with a tPBM helmet for 12 weeks.	tPBM “Neuro” helmet device, with 40 LED diodes (20 red—635 nm + 20 infrared—810 nm) in 20 locations. Average optical power for the 810 nm LED was 52 mW and for the 635 nm LED was 27 mW. Treatment consisted of 12 min of red followed by 12 min of infrared irradiation, giving a total energy of 748.8 J (IR) and 388.8 (red), delivered 6 days per week for 12 weeks (72 total treatments).	tPBM device

## Data Availability

The datasets analyzed in this study are available from the corresponding author upon reasonable request.
